# Function of Cytochrome P450s and Gut Microbiome in Biopesticide Adaptation of *Grapholita molesta* on Different Host Diets

**DOI:** 10.3390/ijms242015435

**Published:** 2023-10-21

**Authors:** Yanjun Liu, Jianmei Yu, Fang Zhu, Zhongjian Shen, He Jiang, Zhen Li, Xiaoxia Liu, Huanli Xu

**Affiliations:** 1 State Key Laboratory of Integrated Management of Pest Insects and Rodents, Institute of Zoology, Chinese Academy of Sciences, Beijing 100101, China; liuyanjun196@163.com (Y.L.);; 2 Department of Entomology, MOA Key Lab of Pest Monitoring and Green Management, College of Plant Protection, China Agricultural University, Beijing 100193, Chinalizhencau@cau.edu.cn (Z.L.);; 3 Institute of Vegetables, Zibo Academy of Agricultural Sciences, Zibo 255000, China; 4 Department of Entomology, Pennsylvania State University, University Park, PA 16802, USA; 5 Institute of Plant Protection, Chinese Academy of Agricultural Sciences, Beijing 100193, China

**Keywords:** *Grapholita molesta*, gut microbiome, cytochrome P450s, emamectin benzoate, xenobiotic adaptation

## Abstract

Insects that feed on various host plants possess diverse xenobiotic adaptations; however, the underlying mechanisms are poorly understood. In the present study, we used *Grapholita molesta*, which shifts feeding sites from peach shoots to apple fruits, as a model to explore the effects of shifts in host plant diet on the profiles of cytochrome P450s and the gut bacteria microbiome, as well as their effects on biopesticide adaptation. We found that the sensitivity of the fruit-feeding *G. molesta* to emamectin benzoate biopesticide was significantly lower than that of the shoot-feeding larvae. We also found that the P450 enzyme activity and the expression of nine cytochrome P450s were enhanced in *G. molesta* fed on Fuji apples compared to those fed on peach shoots. The survival rates of *G. molesta* exposed to emamectin benzoate significantly decreased as each of three of four emamectin benzoate-inducted cytochrome P450 genes were silenced. Furthermore, we discovered the gut bacteria dynamics of *G. molesta* changed with the host shift and the structure of the gut bacteria microbiome was determined by the final diet ingested; additionally, the dysbiosis of the gut microbiota induced by antibiotics could significantly increase the sensitivity to emamectin benzoate. Taken together, our results suggest that the expression of P450s and the composition of the gut bacteria microbiome promote adaptation to emamectin benzoate in *G. molesta*, providing new insights into the molecular mechanisms underlying xenobiotic adaptation in this notorious pest.

## 1. Introduction

Insects are the most abundant animals on Earth, and approximately half of them feed on a variety of host plants. Extensive research on insects suggests that shifts in host plants coincide with distinct patterns of detoxification gene expression and alterations of gut microbiomes [1,2]. For example, multiple detoxification genes and microbiomes were observed to be related to varying insecticide susceptibility in *Spodoptera frugiperda* when fed on different host plants [3]. Therefore, different host plant diets may influence the susceptibility of insects to pesticides through host metabolic genes and symbionts.

The enhanced metabolic detoxification pathway is one of the major mechanisms for insect adaptation to xenobiotics. Insects utilize their enzymatic systems to metabolize pesticides, and resistant populations may possess a higher abundance of detoxification enzymes or enzymes with improved detoxifying capabilities compared to susceptible populations [4]. The cytochrome P450 monooxygenases (CYPs, also called P450s) are the most important large superfamilies of Phase I enzymes in detoxification pathways [5,6]. For example, a brain-specific P450 CYP6BQ9 and other P450s in the CYP6BQ cluster of *Tribolium castaneum* contribute deltamethrin resistance in a resistant *T. castaneum* strain [7,8]. The up-regulation of multiple P450s facilitates adaptation to both insecticide and plant allelochemicals in *Leptinotarsa decemlineata* [9]. Similarly, the sensitivity of two bee species to neonicotinoids is determined by P450s in the CYP9Q subfamily [10]. In *Grapholita molesta*, an insect capable of feeding on multiple hosts, researchers have identified 14 P450s in the CYP2 clade, 30 P450s in the CYP3 clade, 18 P450s in the CYP4 clade, and 15 P450s in the mitochondria clade [11]. These findings suggest that P450 genes likely play important roles in xenobiotic adaptation in *G. molesta*.

Host plant diets not only influence the expression of metabolic genes in hosts, but also modulate the composition of their gut microbiome [3]. The structure of insect gut microbiomes is generally influenced by many endogenous and exogenous factors, including the hosts’ environment [12] and host plants [13,14]. These microbiomes play vital roles by engaging in multiple interactions with hosts, including digestion and nutrition [15,16], metabolism and development of the insect host [1,17], and detoxification of xenobiotics [18]. Gut microbes can be beneficial to herbivores during adaptation to a broad range of hosts. For example, differential profiles of gut microbiota are associated with host shifts in *G. molesta* [19], *Plutella xylostella* [20], and *Leptidea sinapis* [21]. Gut microbes in insects have been linked to insecticide resistance, including the degradation [18,22] and enhancement of host xenobiotic metabolism [23,24], demonstrating their role in modifying xenobiotic adaptation. However, our understanding of insect detoxification genes and gut microbiomes influencing xenobiotic adaptation to various host plant diets in *G. molesta* remains unclear.

The oriental fruit moth *G. molesta* (Lepidoptera: Tortricidae) is a notorious pest in fruit production worldwide, which prefers to feed on the new shoots and fruits of plants within the Rosaceae family, including peach, pear, and apple [25,26,27]. Emamectin benzoate is a biopesticide confirmed to be highly effective against oriental fruit moths [28]. Therefore, we used apple fruit- (AF) and peach shoot (PS)-feeding *G. molesta* and emamectin benzoate as a model system to study the effects of diet shifts on the tolerance of *G. molesta* to emamectin benzoate. In this study, we examined how the diets of *G. molesta*, including AFs and PSs, affect the insect’s P450s and gut microbiome, and their potential roles in *G. molesta*‘s adaptation to emamectin benzoate. The results suggest that host diet-induced biopesticide adaptation is likely influenced by gut microbiome and host metabolic gene expressions. Studying gut microbial communities and gene expression in insects during plant host shifts can enhance our understanding of herbivore ecology and improve the biocontrol of insect pests in agriculture.

## 2. Results

### 2.1. Effects of Host Plant Shifting on the Survival of G. molesta Larvae Exposed to Emamectin Benzoate

**Figure 1:**
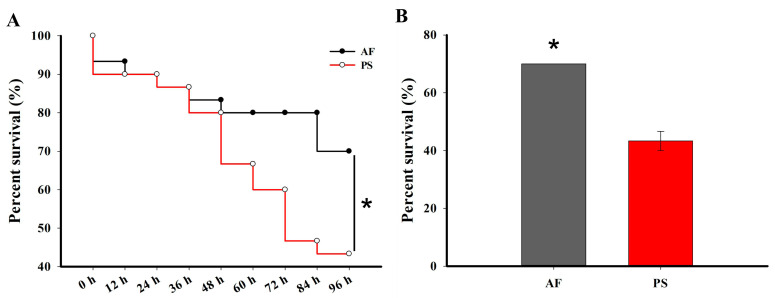
The susceptibility of 5th instar *G. molesta* larvae to emamectin benzoate after feeding on different diets. The survival curves (**A**) and accumulated survival rates (**B**) of 5th instar larvae at 96 h after exposure with 4 mg/L emamectin benzoate in fruit-feeding (AF) and shoot-feeding (PS) *G. molesta*. Survival curves were analyzed by the method of Kaplan–Meier and statistical significance between survival curves was determined using the log-rank test. Each value in panel B is the mean ± SE of three biological replicates (*n* = 20 for each replicate). Statistically significant differences were analyzed using independent Student’s *t*-test, * *p* < 0.05.

According to the results of the bioassay, the LC50 value of emamectin benzoate against *G. molesta* was estimated as 4.00 mg/L (95% CI 3.01–4.84 mg/L). Our results show that the sensitivity of fruit-feeding *G. molesta* larvae (AF) exposed to emamectin benzoate was significantly lower than that of shoot-feeding larvae (PS) (Figure 1A). The accumulated survival rate of shoot-feeding *G. molesta* larvae (PS) after being exposed to emamectin benzoate was 36.5% lower than that of fruit-feeding *G. molesta* (AF) at 96 h (Figure 1B).

### 2.2. Effects of Diets on Regulation of P450 Enzyme Activities and Gene Expression in G. molesta Larvae

**Figure 2:**
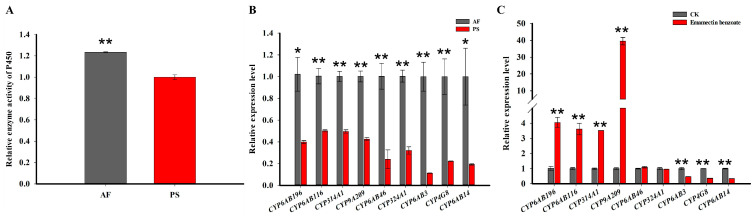
P450 enzyme activities and expression profiles of P450 genes under different treatments. (**A**) The P450 enzyme activities in 5th instar larvae fed on Fuji apples (AF) or peach shoots (PS). (**B**) The expression levels of nine P450s in 5th instar larvae fed on Fuji apples (AF) or peach shoots (PS). (**C**) The differential expression of nine P450 genes in *G. molesta* with or without emamectin benzoate exposure. Each value is the mean ± SE of three biological replicates (*n* = 5 for each replicate). Statistically significant differences were analyzed using independent Student’s *t*-test, * *p* < 0.05; ** *p* < 0.01.

As is well known, P450-mediated detoxification is crucial for adapting to various xenobiotics, including pesticides and host plant allelochemicals [7,8,9]. Therefore, we hypothesized that the promotion of biopesticide adaptation in *G. molesta* with a shift in host plants may occur through the regulation of P450-mediated detoxification. To test our hypothesis, we first compared the P450 activities between fruit-feeding and shoot-feeding *G. molesta* larvae. We found that the activities of P450 enzymes in the fruit-feeding *G. molesta* (AF) were significantly elevated compared with the shoot-feeding *G. molesta* (PS) (Figure 2A). In order to identify which P450(s) may contribute biopesticide adaptation, we performed a transcriptome analysis (unpublished) of gene expression between fruit-feeding and shoot-feeding *G. molesta* larvae. Nine P450 genes (*CYP6AB196*, *CYP6AB116*, *CYP314A1*, *CYP9A209*, *CYP6AB46*, *CYP324A1*, *CYP6AB3*, *CYP4G8*, and *CYP6AB14*) were significantly up-regulated in fruit-feeding *G. molesta* larvae compared to the shoot-feeding *G. molesta* larvae (Figure 2B). Among these nine P450s, four of them were induced after exposure to emamectin benzoate (LC50, 4.00 mg/L) in the *G. molesta* larvae fed on an artificial diet (Figure 2C), indicating these four P450s may play roles in the adaptation to emamectin benzoate.

### 2.3. Effects of P450 Gene Silencing on the Sensitivity of G. molesta Exposed to Emamectin Benzoate

**Figure 3:**
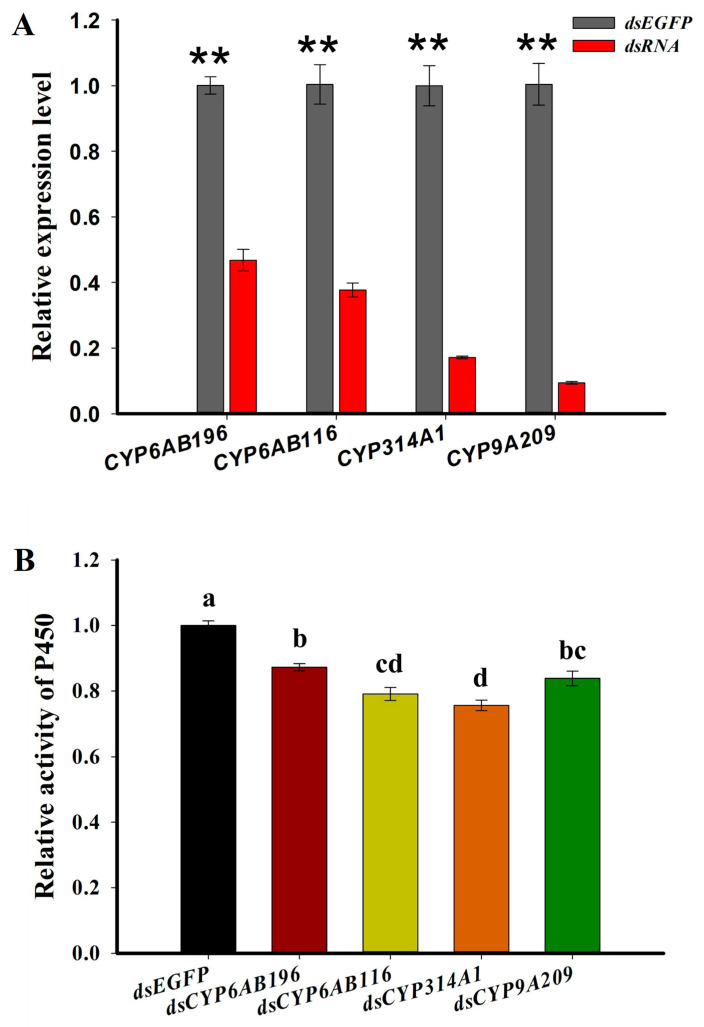
The RNA interference efficiency on P450 gene expression (**A**) and enzyme activity (**B**) after dsRNA injection. RNA samples were collected from the 5th instar larvae fed on artificial diet at 24 h following dsRNA injection. Each value is the mean ± SE of three biological replicates (*n* = 5 for each replicate). Statistically significant differences were analyzed using independent Student’s *t*-test, ** *p* < 0.01. The letters above each bar indicate significant differences according to one-way ANOVA analysis, followed by a Tukey’s HSD multiple comparison test (*p* < 0.05).

**Figure 4:**
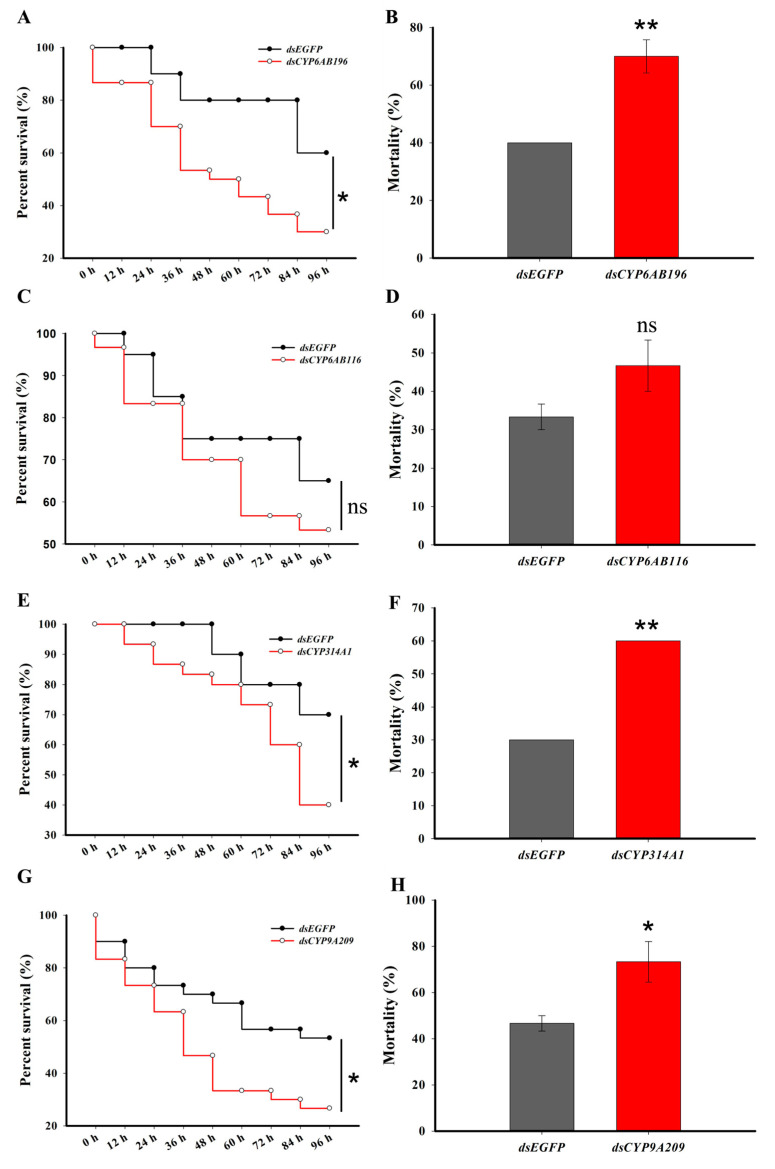
Effects of P450 genes knockdown on the sensitivity to emamectin benzoate (4.00 mg/L) in *G. molesta*. Survival curves of 5th instar larvae fed on artificial diet exposed with emamectin benzoate after the knocking down of *CYP6AB196* (**A**), *CYP6AB116* (**C**), *CYP314A1* (**E**) and *CYP9A209* (**G**). Survival curves were analyzed by the method of Kaplan–Meier and statistical significance between survival curves was determined using the log-rank test, * *p* < 0.05. The mortality of larvae exposed to emamectin benzoate for 96 h after *CYP6AB196* (**B**), *CYP6AB116* (**D**), CYP314A1 (**F**) and *CYP9A209* (**H**) knockdown. Each value is the mean ± SE of three biological replicates (*n* = 20 for each replicate). Statistically significant differences were analyzed using independent Student’s *t*-test, * *p* < 0.05; ** *p* < 0.01; ns, an abbreviation for “not significant”.

To further investigate the roles of these four target P450 genes in emamectin benzoate adaptation, we knocked down these P450s by RNAi, one by one. The relative expression levels of the four genes significantly decreased 24 h after dsRNA injection compared to EGFP dsRNA injection: 53.3% for *CYP6AB196*, 62.4% for *CYP6AB116*, 82.8% for *CYP314A1*, and 90.6% for *CYP9A209* (Figure 3A). The enzymatic activities of each of the P450s were also significantly decreased in all silenced samples compared to the control (Figure 3B), indicating the effectiveness of the RNAi. To investigate the functions of these four P450s in *G. molesta*’s emamectin benzoate adaptation, we tested the sensitivity of *G. molesta* larvae to 4.00 mg/L emamectin benzoate after knocking down each of these four genes. After silencing *CYP6AB196*, *CYP314A1* or *CYP9A209*, the susceptibilities of larvae exposed to emamectin benzoate were significantly enhanced (Figure 4A,E,G). The accumulated mortalities of *G. molesta* larvae after *CYP6AB196*, *CYP314A1* or *CYP9A209* knockdown exposed with emamectin benzoate at 96 h were increased by 42.9%, 50.0% and 36.4%, respectively, compared to controls (Figure 4B,F,H). However, there was no statistical difference in the sensitivity to emamectin benzoate after the knockdown of *CYP6AB116* compared to the control, although the mortality was increased (Figure 4C,D). The percentages of mortality between non-injected and dsEGFP-injected groups were not significantly different [29].

### 2.4. Analysis of 16S rRNA Sequencing

Microbial community compositions of 30 gut samples from 10 groups (AF3, AF4, AF5, PS3, PS4, PS5, AF_PS4, AF_PS5, PS_AF3, and PS_AF5) were obtained by the MiSeq sequencing method (abbreviations: AF3, AF4, and AF5 stand for third, fourth, and fifth instar larvae fed on Fuji apples, respectively; PS3, PS4, and PS5 stand for third, fourth and fifth instar larvae fed on peach shoots, respectively; AF_PS4 and AF_PS5 stand for third instar larvae fed by Fuji apples transferred to peach shoots until fourth or fifth instars, respectively; PS_AF4 and PS_AF5 stand for third instar larvae fed on peach shoots transferred to Fuji apples until fourth or fifth instars, respectively). Sequencing data were uploaded to the NCBI Sequence Read Archive (SRA) BioProject PRJNA695398. After demultiplexing analysis, a total of 3279 operational taxonomic units (OTUs) were obtained (Table S2). The number of sequences for each sample was normalized to 15,963, and the rarefaction curves of Shannon index rarefaction curves reflected a saturated sampling depth (Figure S1). Sequencing integrity was determined by Good’s coverage. The coverage ranged from 98.56 to 99.82%, suggesting that the great majority of species present in the samples were successfully identified in the current study (Table S2). A ribosomal database was used to classify sequences. All bacteria identified were classified into 49 phyla, 121 classes, 257 orders, 469 families and 1028 genera.

### 2.5. Comparison of the Gut Microbiota of G. molesta Fed on Different Diets

**Figure 5:**
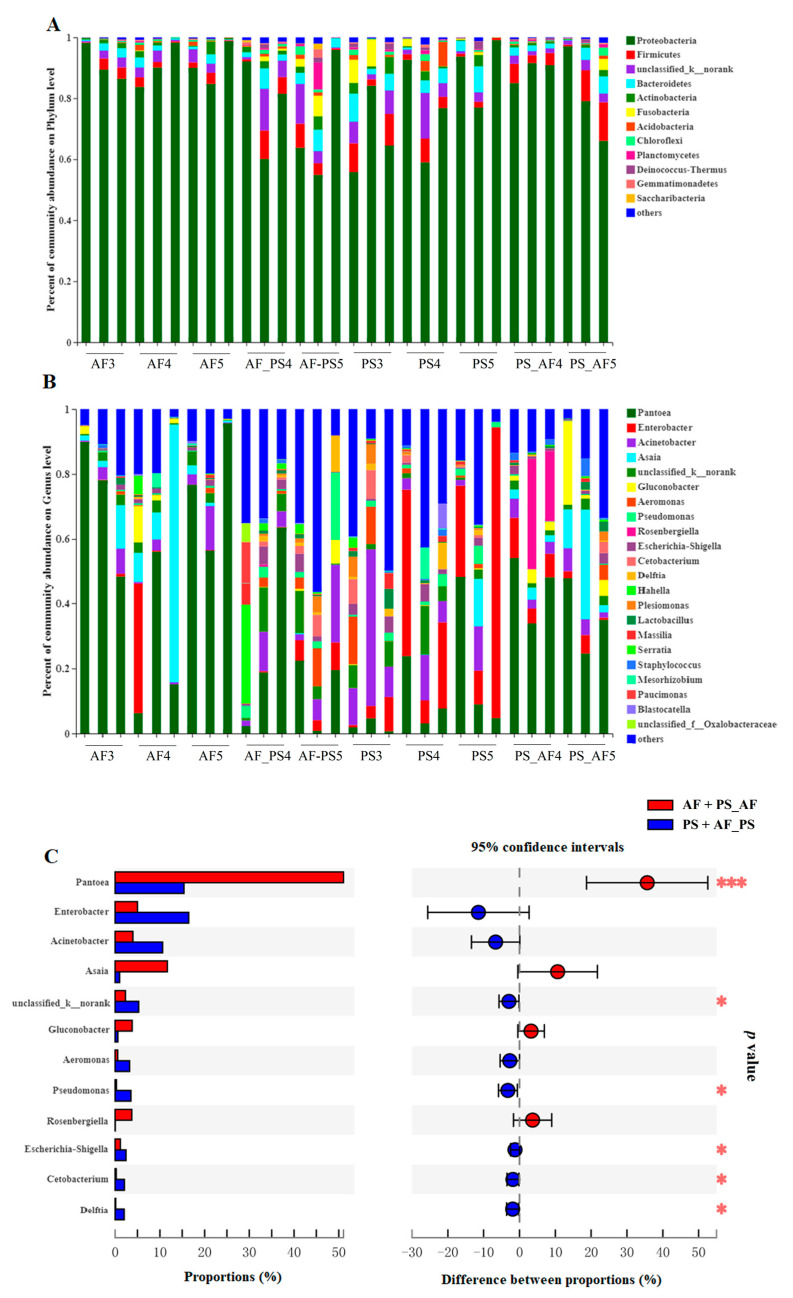
Relative abundance of bacterial composition at the phylum (**A**) and family levels (**B**) and top 12 genera between fruit-feeding *G. molesta* and shoot-feeding *G. molesta* (**C**). Abbreviations: AF3, AF4, and AF5 stand for 3rd, 4th, or 5th instar larvae fed on Fuji apples, respectively. PS3, PS4, and PS5 stand for 3rd, 4th, or 5th instar larvae fed on peach shoots, respectively. AF_PS4 and AF_PS5 stand for 3rd instar larvae fed by Fuji apples transferred to peach shoots until 4th or 5th instars, respectively. PS_AF4 and PS_AF5 stand for 3rd instar larvae fed on peach shoots transferred to Fuji apples until 4th or 5th instars, respectively. Taxa with an abundance <1% (**A**) and 5% (**B**) were included in “others”. Welch’s *t*-test was used to evaluate the differences. Significant differences (* *p* < 0.05; *** *p* < 0.001) are highlighted in red (**C**).

The variations in gut microbiota between and within each species are reflected in the alpha diversities (Table S2). In general, AF_PS5 exhibited the highest bacterial richness and diversity, whereas PS5 held the lowest richness values and AF5 had the lowest diversity indices. There were no significant differences in the ACE and Chao1 indices (Table S2). Gut microbiota were identified at different taxonomic levels. The relative abundances of major phyla were commonly observed across all the samples (Figure 5A). Those sequences that could not be assigned to known microbial phyla were grouped as “unclassified_k_norank”, which represented 3.78% of the entire data set. A few phyla occurred at low abundance and sporadically in some samples and were referred to as “others” (1% of the total sequences). At the phylum level, samples mainly contained Proteobacteria (mean ± SD = 82.71% ± 2.54% of total sequences), Firmicutes (4.03% ± 0.65%), Bacteroidetes (3.1% ± 0.45%) and Actinobacteria (1.57% ± 0.27%). Proteobacteria was the most dominant phylum in the gut samples of *G. molesta*, especially in fruit-feeding *G. molesta* larvae. To visualize the dynamic patterns of gut bacteria in the diet-switching *G. molesta*, a column chart in the genera level was constructed for the datasets (Figure 5B). Among samples, Pantoea in the phyla Proteobacteria was the most dominant genus, especially in the fruit-feeding (AF) (58.06% ± 10.43%) and shoot-feeding transfer to fruit-feeding (PS_AF) (40.37% ± 4.7%) samples. The abundance of Pantoea in fruit-feeding (AF) and shoot-feeding transfer to fruit-feeding (PS_AF) was significantly higher than that in shoot-feeding (PS) and fruit-feeding transfer to shoot-feeding (AF_PS), whereas Pseudomonas was enriched in PS and AF_PS compared to AF and PS_AF (Figure 5C).

**Figure 6:**
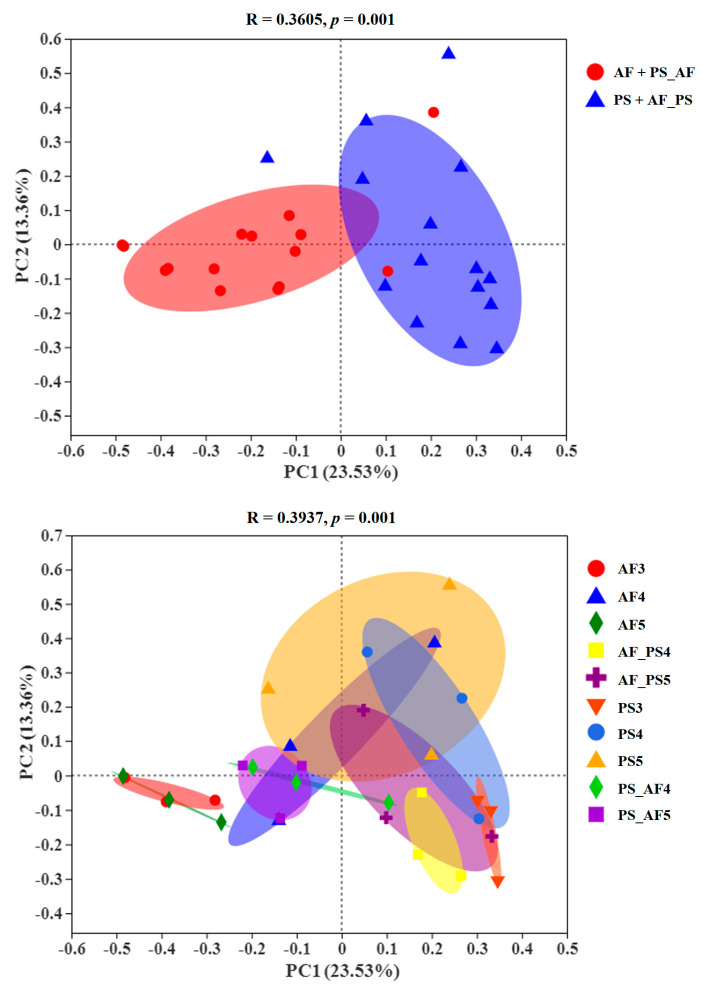
Principal coordinate analysis (PCoA) visualization using the Bray–Curtis dissimilarity measurement separating samples. Principal coordinate (PCoA) analysis based on Bray–Curtis distance. Abbreviations: AF3, AF4, AF5 stand for 3rd, 4th, 5th instar larvae fed on Fuji apples. PS3, PS4, PS5 stand for 3rd, 4th, 5th instar larvae fed on peach shoots. AF_PS4, AF_PS5 stand for 3rd instar larvae fed by Fuji apples transferred to peach shoots until 4th or 5th instars. PS_AF4, PS_AF5 stand for 3rd instar larvae fed on peach shoots transferred to Fuji apples until 4th or 5th instars.

Similarities in the microbial community compositions among samples were compared by PCoA based on Bray–Curtis (Figure 6). In the scatter plot, the first two principal coordinates, PCO1 and PCO2, explained 23.53% and 13.36% of the data variation, respectively. The ANOSIM analysis revealed significant differences in the structures (ANOSIM, R = 0.3605 for two groups, R = 0.3937 for ten groups, *p* = 0.001) of the gut microbiota among different groups. The microbiota of fruit-feeding (AF) and shoot-feeding transfer to fruit-feeding (PS_AF) *G. molesta* were clustered closely. Equally, microbiota of shoot-feeding (PS) and fruit-feeding transfer to shoot-feeding (AF_PS) *G. molesta* were clustered closely.

### 2.6. Roles of Gut Microbiota on Survival of G. molesta Larvae Exposed to Emamectin Benzoate

To determine the potential roles of *G. molesta* gut microbiota in emamectin benzoate adaptation, antibiotics were used to eliminate the gut microbiota from larvae. The efficacy of elimination of gut bacteria was confirmed by plating gut homogenates onto LB agar plates (Figure S2A) and performing PCR (Figure S2B) and qPCR (Figure S2C) analysis using bacterial 16S rRNA gene universal primers (Table S1). Compared with the control, far fewer colonies were found in the plates with gut microbiota from larvae treated with 100 mg/L, 200 mg/L, and 400 mg/L of antibiotics. No colonies were found in the plate with gut suspensions from 800 mg/L antibiotic-treated larvae (Figure S2A). PCR and qPCR showed that after being treated with antibiotics, there was almost no 16S rRNA detected (Figure S2B,C). However, the antibiotic cocktail applied at 100 mg/L and 200 mg/L had no significant effects on the survival of *G. molesta* (Figure S2D). In contrast, concentrations of 400 mg/L and 800 mg/L caused significantly higher mortalities than the control (Figure S2D). Therefore, we chose the 200 mg/L antibiotic treatment to assess the survival and P450s enzyme activities in *G. molesta* exposed to emamectin benzoate.

**Figure 7:**
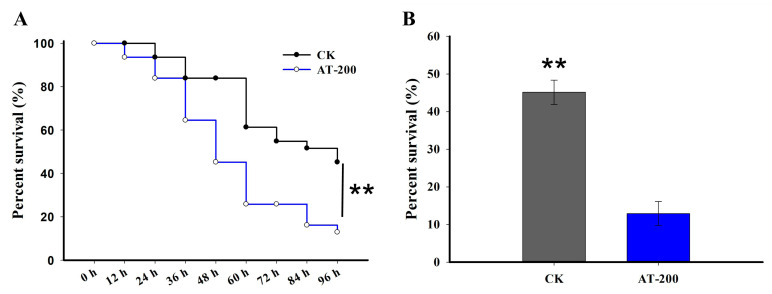
The susceptibility of 5th instar *G. molesta* larvae to emamectin benzoate under antibiotic treatment. The survival curves (**A**) and accumulated survival rates (**B**) at 96 h exposure to emamectin benzoate in 200 mg/L antibiotic-treated (AT-200) and no antibiotic (CK)-treated 5th instar *G. molesta* larvae. Survival curves were analyzed by the method of Kaplan–Meier and statistical significance between survival curves was determined using the log-rank test. Each value is the mean ± SE of three biological replicates (*n* = 62 for all three replicates). Statistically significant differences were analyzed using independent Student’s *t*-test, ** *p* < 0.01.

Following exposure to 200 mg/L antibiotics, *G. molesta* exhibited significantly increased sensitivity to emamectin benzoate in the antibiotic-treated group (AT-200) compared to the control group (CK) (Figure 7A). After 96 h of treatment, the accumulated survival rate of the antibiotic-treated group (AT-200) was significantly decreased by 36.93% compared to the control group (CK) (Figure 7B).

## 3. Discussion

The tolerance of insects to insecticides can be influenced by their diets, including plant species and tissue they feed on. Emamectin benzoate is a highly effective biopesticide for controlling *G. molesta* [28]. In our study, we observed significantly higher larval survival rates in fruit-feeding *G. molesta* exposed to emamectin benzoate compared to shoot-feeding *G. molesta*. This result suggests that the emamectin benzoate detoxification ability of *G. molesta* feeding on fruits was different from that of those feeding on shoots.

P450 enzymes are integral membrane-bound hemoproteins that play a pivotal role in the detoxification of xenobiotics and the maintenance of homeostasis [10]. To further investigate the mechanisms of response varying the susceptibility to emamectin benzoate, we assessed both the relative detoxification enzyme activities of P450s and the relative expression of P450 genes. The activity of the P450 enzyme was significantly increased in the fruit-feeding *G. molesta* compared to those feeding on shoots. Moreover, nine P450 genes were found to be up-regulated in fruit-feeding *G. molesta* by RNA-seq and qRT-PCR (Figure 2). Furthermore, following the silencing of three out of four P450 genes by RNAi, there was a significant decrease in the percentage of larval survival in response to emamectin benzoate (Figure 3). A similar pattern was observed in *S. frugiperda* [3] and *Trialeurodes vaporariorum* [30], where insecticide tolerance was linked to metabolism P450 gene expression and detoxification enzyme activities, influenced by feeding on different host plants. Our findings suggest that *G. molesta*’s tolerance to insecticides is influenced by its host diet, potentially due to enhanced detoxification enzyme activities and the differential expression of xenobiotic metabolism-associated genes.

Insects’ gut microbes play important roles in their interactions with insect hosts, facilitating the adaptation of insects to their environment [1,16]. Dynamic changes of gut microbiome composition and activity associated with shifts in diet provide the basis for the insect host to rapidly adapt to their environment and ensure their survival [1]. *G. molesta* is a global pest of stone and pome fruits, with the ability to shift its diet between shoots and fruits of various plant species during its development [19,31]. Our recent studies have revealed significant variations in the gut microbiota of *G. molesta* larvae when they consume shoots and fruits from different plant species, highlighting the rapid adaptability of the gut microbiome to dietary changes [31]. In the current study, we found that the gut bacteria of the fruit-feeding group and the shoot transfer to the fruit-feeding group clustered closely, while the shoot-feeding group and the fruit transfer to shoot-feeding group clustered closely (Figure 6). These findings indicate that the structure of the gut microbiome was influenced by the final diet consumed. Our results align with previous research on gut bacteria in *Bombyx mori*, *Diabrotica virgifera virgifera*, and *Helicoverpa armigera* [1,2,32], demonstrating that the gut microbiota of *G. molesta* contribute to the rapid adaption to the external environment.

Given that bacteria harbored in the gut of *G. molesta* facilitate rapid adaptation, we hypothesize that gut bacteria play pivotal roles in the detoxification capability of *G. molesta* to emamectin benzoate during diet shift. In our study, we assessed the susceptibility of *G. molesta* to emamectin benzoate following antibiotic treatment, a classical method for disturbing or removing gut bacteria to investigate gut bacteria function [33]. Our results show that the mortalities of *G. molesta* exposed to emamectin benzoate were elevated after antibiotic treatment (Figure 7), suggesting the gut microbiome contributes to pesticide resistance and potentially plays a role in adaptation. Similar findings have been reported in *Bactrocera dorsalis*, *Anopheles stephensi*, *Apis mellifera* and *B. mori* [18,23,34,35].

Previous research has shown that gut bacteria play important roles in adaptation to new plant hosts and can be involved in pesticide detoxification, contributing to insecticide resistance [18,34,35]. Under some circumstances, gut bacteria can contribute pesticide resistance by directly degrading chemicals [36]. However, this is not the case in many Lepidoptera insects [35,37]. Moreover, gut microbiota can indirectly influence insect host resistance by affecting the relationship between gut bacteria and the host’s detoxification abilities more broadly [23,24,38]. The gut microbiota may play indirect roles to enhance the host’s xenobiotic metabolism. For example, it has been proposed that gut microbiota can enhance the host’s detoxification capability by regulating insect cytochrome P450 enzyme activities in *Nilaparvata lugens* [24]. We offer insights into microbiome modulation that may play a role in pesticide resistance. However, the precise mechanisms and relationships between the microbiome and the metabolic genes of the insects or microbes require further investigation.

In this study, we revealed that different host diets affected *G. molesta*’s tolerance to emamectin benzoate, and the variation in biopesticide susceptibility was attributed to differences in the expression of P450 genes. In addition, we found that the composition of gut bacteria rapidly shifts with varying host diets, potentially playing pivotal roles in both adaptation to natural environments and pesticide tolerance in agricultural settings. In conclusion, our research revealed the gut bacteria and insect P450 activity play important roles in plant host shift events of *G. molesta* and its sensitivity to emamectin benzoate, providing insights into the functions of gut bacteria and detoxification capabilities in *G. molesta*. Recognizing host and microbiota interactions may provide valuable information in the generation of tools for pest resistance management and for developing pest control strategies.

## 4. Materials and Methods

### 4.1. Insect Maintenance and Sample Collection

The *G. molesta* were reared in the Integrated Pest Management laboratory of China Agricultural University under laboratory conditions at 25 ± 1 °C and 50 ± 10% RH, with a 15 h:9 h light/dark photoperiod. Plastic boxes (15 × 10.2 × 8.5 cm) were used for adults laying eggs (the adults grew from the larva fed on artificial diet). Then the neonates were reared on fresh Fuji apples (AF) or peach shoots (PS). Fuji apples and shoots of peach trees (*Prunus persica* (L.)) were collected from Beijing, China.

The samples of AF3, AF4, and AF5 stand for 3rd, 4th, and 5th instar larvae fed on Fuji apples, respectively. PS3, PS4, and PS5 represent 3rd, 4th, and 5th instar larvae fed on peach shoots, respectively. The samples of AF_PS4 and AF_PS5 indicate 3rd instar larvae fed on Fuji apples transferred to peach shoots until 4th and 5th instar, respectively. PS_AF4 and PS_AF5 stand for 3rd instar larvae fed on peach shoots transferred to Fuji apples until 4th and 5th instar, respectively.

### 4.2. Bioassay of G. molesta with Emamectin Benzoate

To estimate the LC50 of emamectin benzoate (the concentration that causes mortality in 50% of the *G. molesta* population), 5th instar larvae fed on artificial diet were soaked in 1–10 mg/L of emamectin benzoate (technical grade 92%; Weiyuan Biological Ltd., Shijiazhuang, China) dissolved with 0.1% Triton X-100 (Beijing Solar Bio Science and Technology Co., Ltd., Beijing, China) for 5 s. The mortality was determined by counting the number of dead insects in each group every 12 h until 96 h. Equal 5th instar larvae treated with 0.1% Triton X-100 solution were counted as control. Three replicates were conducted with at least 15 adults for each replicate.

In order to determine the effects of different diets on the sensitivity of *G. molesta* to emamectin benzoate, the 5th instar larvae of the same size were selected for bioassay from *G. molesta* feeding on the fruits and shoots. The concentration of emamectin benzoate used in the test was LC50 and the bioassay procedure was the same as above. Each treatment contained three replicates and at least 15 larvae were used for each replicate.

### 4.3. P450 Gene Expression and P450 Enzyme Activity Feeding on Different Host Diets

In both fruit-feeding *G. molesta* (AF) and peach shoot-feeding *G. molesta* (PS) groups, we collected ten 5th instar larvae. In the treatment of antibiotics, ten 5th instar larvae fed on an artificial diet with antibiotic or sterile water added (control) were collected. For emamectin benzoate treatment, ten 5th instar larvae soaked in LC50 of emamectin benzoate dissolved in 0.1% Triton X-100 or 0.1% Triton X-100 alone (control) for 5 s were collected after 24 h for RNA extraction. Total RNA was extracted from larvae samples using TRIzol reagent (TaKaRa, Kyoto, Japan). Then, cDNA was synthesized from 1 μg of total RNA using a PrimeScript RT reagent kit with gDNA Eraser (TaKaRa, Kyoto, Japan). Nine P450 genes (*CYP6AB196*, *CYP6AB116*, *CYP314A1*, *CYP9A209*, *CYP6AB46*, *CYP324A1*, *CYP6AB3*, *CYP4G8*, and *CYP6AB14*) were obtained from our unpublished *G. molesta* transcriptome study. Primers were synthesized by Sangon Biotechnology Co., Ltd. (Shanghai, China) (Table S1). Actin (KF022227.1) [39] and glyceraldehyde-3-phosphate dehydrogenase (GAPDH, KJ094948.1) [29,40] were used as reference genes for qRT-PCR. The quantitative analysis was performed using the 2-ΔΔCt method [41]. Three biological replications were conducted independently.

The activity of P450 enzymes was assayed using a P450 Elisa assay kit (Enzymelink Biological Ltd., Shanghai, China) according to the manufacturer’s instructions. These assays were conducted with a SPECTRA max GEMINI XS spectrofluorometer (Molecular Devices, Baltimore, MD, USA) at 450 nm. Each treatment contained three replicates and each replicate had at least 10 larvae.

### 4.4. RNA Interference (RNAi)

RNAi technology was used to detect the effects of target P450 genes on the sensitivity of *G. molesta* to emamectin benzoate. Double-stranded RNAs (dsRNAs) were synthesized using a MEGAscript RNAi kit (Ambion, Fremont, CA, USA). The dsRNA targets of four target P450 genes are shown in Figure S3. In the injections, 3 μg of dsRNAs were injected into the proleg of 5th instar larvae using a capillary microsyringe. The controls were injected with an equal amount of EGFP dsRNAs. At least 10 larvae were randomly collected at 24 h after dsRNA injection to test the P450 enzyme activity and efficiency of gene silencing using qRT-PCR.

In the survival assay, after injecting 3 µg dsRNA for 12 h, the larvae were immersed in LC50 of emamectin benzoate for 5 s and then the number of dead larvae was counted every 12 h up to 96 h. Larvae injected with dsEGFP were used as the controls. At least 15 individuals were used in each group and each group contained three replicates.

### 4.5. DNA Extraction and High-Throughput Sequencing

#### 4.5.1. DNA Extraction

To isolate bacteria from the gut of *G. molesta* larvae, samples of AF, PS, AF_PS, and PS_AF were collected and then surface-sterilized in 75% ethanol for 90 s, followed by three rinses in sterile water. Gut dissection was conducted with sterile phosphate-buffered saline (PBS) under a stereomicroscope. At least 15 guts were pooled as a replication and each treatment had three replications. Total DNA from each sample was extracted using Insect DNA kit (OMEGA, Dallas, TX, USA) according to the instructions. DNA quantity and quality were measured using a NanoDrop 2000 spectrophotometer (Thermo Fisher Scientific, Waltham, MA, USA). DNA integrity was determined using 1% agarose gel.

#### 4.5.2. PCR Amplification and High-Throughput Sequencing

The V3-V4 variable region of the 16S ribosomal RNA (rRNA) gene was amplified using 341F (5′ CCTACGGGNGGCWGCAG 3′) and 805R (5′ GACTACHVGGGTATCTAATCC 3′) primers [33]. PCR amplification mixture contained 15 μL of 2 × Taq Master Mix, 1 μL of 10 μM Bar-PCR primer F, 1 μL of 10 μM primer R, and 10–20 ng of genomic DNA. The PCR cycles were as follows: 94 °C for 3 min followed by 5 cycles at 94 °C for 30 s, 45 °C for 20 s, and 65 °C for 30 s, then 20 cycles of 20 s at 94 °C, 20 s at 55 °C and 30 s at 72 °C, and a final extension at 72 °C for 5 min. Illumina bridge-type compatible PCR primers were introduced for the second round of PCR amplification as follows: 95 °C for 30 s; followed by 5 cycles at 95 °C for 15 s, 55 °C for 15 s and 72 °C for 30 s, and finally 5 min at 72 °C. The obtained PCR products were purified by the Agencourt AMPure XP beads (Beckman, Tustin, TX, USA). The 16S rRNA gene amplicons were quantified with 10 ng and subjected to pyrosequencing using the Illumina MiSeq PE300 platform (Sangon Biotech Co., Ltd., Shanghai, China).

#### 4.5.3. Bioinformatics Analysis

Raw fastq files were demultiplexed, quality-filtered by Trimmomatic and merged by FLASH (version 1.2.11) software. We used UCHIME for identifying and removing the chimera sequence [42]. Low-quality sequences, chloroplasts and mitochondria were removed prior to further analyses. Clean sequences were clustered into Operational Taxonomic Units (OTUs) based on a 97% identity threshold using UPARSE (version 7.1). The sequences were annotated and blasted against SILVA 16S rRNA database version 138 using the RDP Classifier algorithm. Finally, the number of sequences per sample was normalized to 15,963 (the smallest number of final, quality-controlled reads among the samples). A representative sequence from each OTU was selected for downstream analysis. The Shannon rarefaction curves and other richness and diversity indices of bacterial community (i.e., ACE, Chao1, Shannon and Simpson) were estimated using the Mothur software (version 1.46.1) [43]. Welch’s *t*-test was utilized to identify changes with significant differences between two different groups. Principal coordinate analysis (PCoA) based on the Bray–Curtis similarities index was applied to rank the bacterial communities. Analysis of similarity (ANOSIM) was performed to determine the differences among groups.

### 4.6. Antibiotic Assay

The 3rd instar larvae were surface-sterilized with 75% ethanol for about 1 min, washed with distilled water three times, then transferred to artificial diet containing rifampicin, penicillin, tetracycline and streptomycin, and reared for about 5 d until the 5th instar. The concentration of antibiotic cocktail was 100 mg/L, 200 mg/L, 400 mg/L, and 800 mg/L, respectively. The control 3rd instar larvae were fed with an artificial diet added with an equal amount of sterile water until the 5th instar.

To investigate the effects of antibiotics on gut microbiota, 10 antibiotic-treated and untreated larvae were randomly selected and dissected aseptically, respectively. The homogenates of the guts were serially diluted 1000 times with PBS and plated onto Luria–Bertani medium (LB) agar plates. The plates were incubated at 30 °C for 2 to 3 d. At the same time, PCR and qPCR were also used to check the efficacy of elimination of gut bacteria using bacterial 16S rRNA gene universal primers (Table S1). The qPCR was performed in a reaction volume of 20 μL containing 1 μL DNA, 10 μL SYBR Green Supermix (TaKaRa, Kyoto, Japan), 2 μL primers (Table S1), and 7 μL ddH2O using Bio-Rad CFX Connect™ Real-Time PCR System (Bio-Rad, Hercules, CA, USA) with the procedure as follows: initial denaturation at 95 °C for 3 min, followed by 35 cycles of 10 sec at 95 °C, 30 sec at 55 °C, and 1 min at 72 °C.

The number of dead larvae fed with different antibiotic dosages was recorded. The larvae fed with antibiotic at 200 mg/L and non-antibiotic diets were selected to detect the effects of the gut microbiota on the sensitivity of *G. molesta* to the LC50 of emamectin benzoate. The process was the same as above, and the mortalities were recorded by counting the number of dead insects in each group every 12 h up to 96 h. The number of larvae used for each replicate in each group was at least 15. Three replicates were performed for each group.

### 4.7. Statistical Analysis

Software SPSS20.0 (SPSS Inc., Chicago, IL, USA) was used to perform statistical analysis. Data were expressed as the mean ± standard error (SE) of three independent replicates. Student’s *t*-test was used to analyze differences between two experimental treatments. Differences among multiple treatments were analyzed by one-way ANOVA, followed by a Tukey’s HSD multiple comparison test. In all tests, significance levels are denoted by * (0.01 < *p* < 0.05), ** (*p* < 0.01) and *** (*p* < 0.001). Survival curves were analyzed by the method of Kaplan–Meier and statistical significance between survival curves was determined using the log-rank test (*p* < 0.05).

## Data Availability

Not applicable.

## Supplementary Materials

The following supporting information can be downloaded at: https://www.mdpi.com/article/10.3390/ijms242015435/s1.
