# Supplementary material for: Function of Cytochrome P450s and Gut Microbiome in Biopesticide Adaptation of Grapholita molesta on Different Host Diets
Source: Int J Mol Sci. 2023 Oct 21;24(20):15435. doi: 10.3390/ijms242015435 (PMC10607806; doi:10.3390/ijms242015435)
Supplement: Supplementary file 1 [file ijms-24-15435-s001.zip › ijms-2640220-supplementary.pdf]

Supplementary Materials:

Figure S1. Shannon rarefaction curves for all samples.

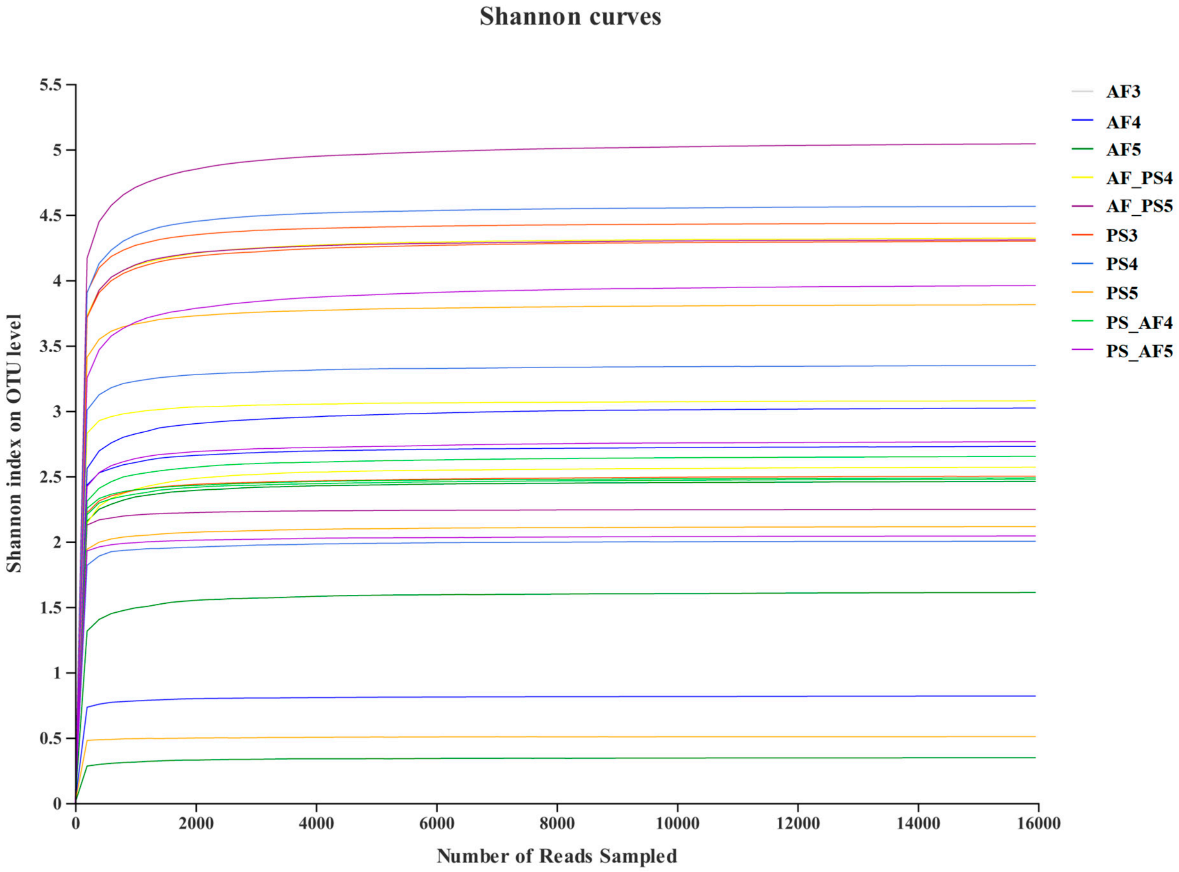

Figure S2. Effects of different antibiotic dosage treatments (0, 100, 200, 400 or 800 mg/L) to eliminate gut microbiota and on the survival of *G. molesta*. The efficacy of elimination of gut bacteria confirmed by (A) culturing gut homogenates on LB agar plates, (B) by performing PCR analysis and (C) by performing qPCR analysis. The mortality of *G. molesta* treated with antibiotic (D). Letters above each group indicate significant differences (one-way ANOVA, Turkey post-hoc test, $p < 0.05$) in the mean values.

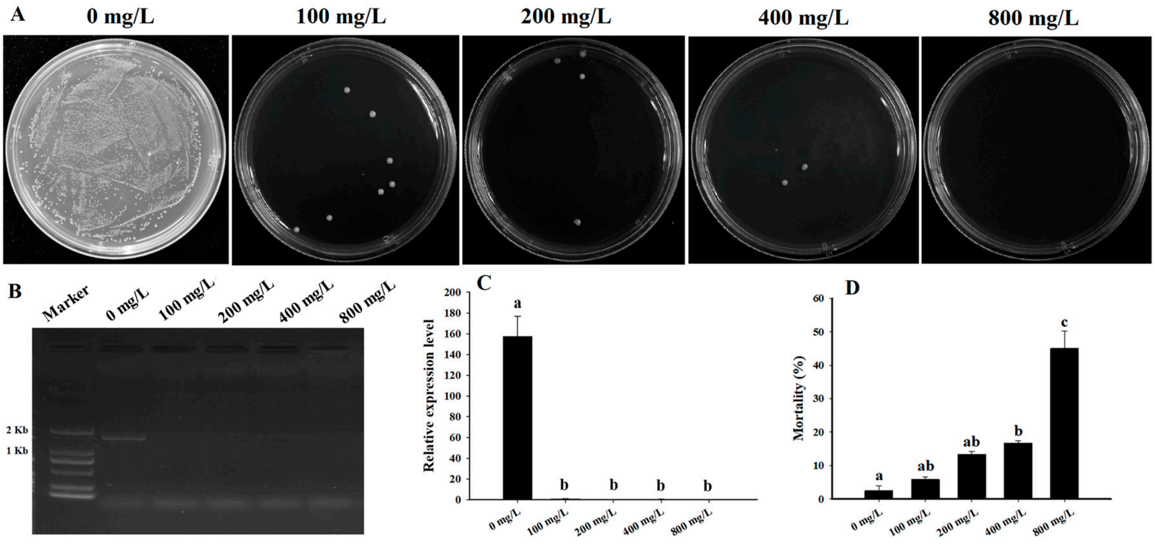

Figure S3. Nucleotide sequence of four target P450 genes. Primer targets for qRT-PCR were marked with pink shading, while those for dsRNA synthesis were marked with yellow shading.

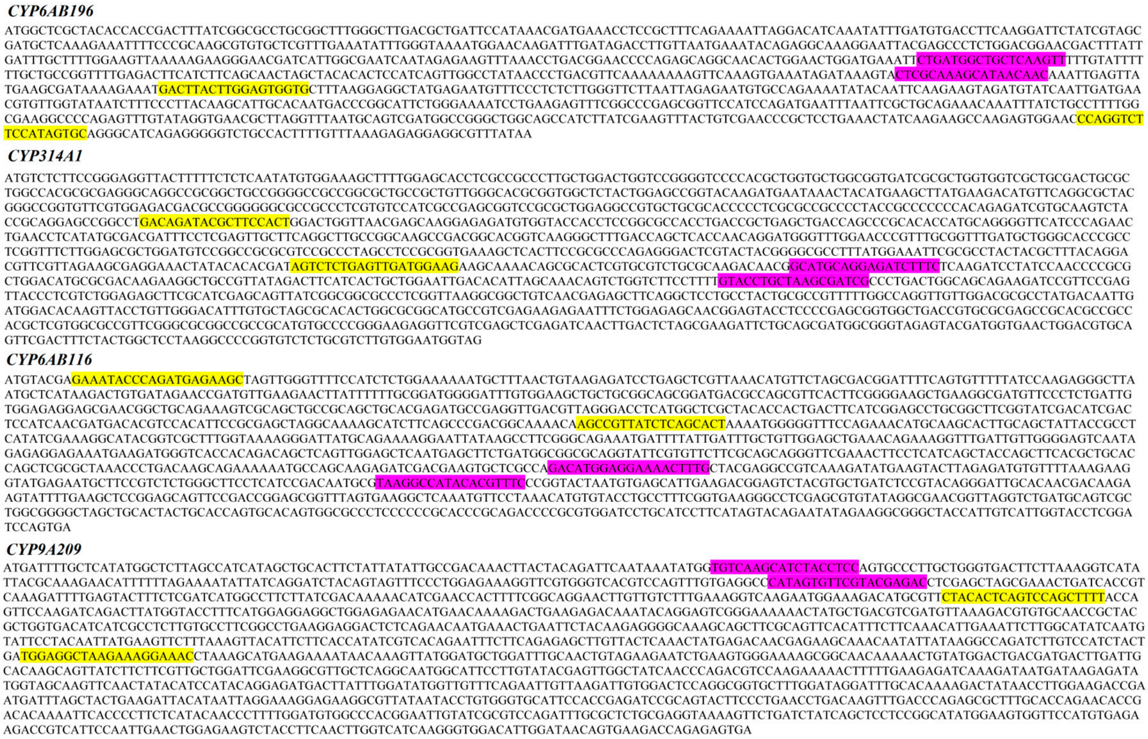

Table S1. Primers used in the current study.

Table S1.

Primer name	Primer sequence (5'-3')
16S-27F	AGAGTTTGATCCTGGCTCAG
16S-1492R	TACGGYTACCTTGTTACGACTT
16SF	TCCTACGGGAGGCAGCAGT
16SR	GGACTACCAGGGTATCTAATCCTGTT

CYP6AB196-ORF-Forward	CCTCATGGCTCGCTACACC
CYP6AB196-ORF-Reverse	CTGGAGCAACTTATCGTTTGC
CYP6AB116-ORF-Forward	GATGTACGAGAAATACCCAG
CYP6AB116-ORF-Reverse	GTCACTGGATCCGAGGTAC
CYP314A1-ORF-Forward	GTTCCTCCTGTCAGTCTT
CYP314A1-ORF-Reverse	TGAGAGATCTGGTAAACGA
CYP9A209-ORF-Forward	GTACTCGTAGCCTAAGTGG
CYP9A209-ORF-Reverse	TCACTCTCTGGTCTTCACT
CYP6AB196-qPCR-Forward	CTGATGGCTGCTCAAGTT
CYP6AB196-qPCR-Reverse	GTTGTTATGCTTTGCGAG
CYP6AB116-qPCR-Forward	GACATGGAGGAAAACTTTG
CYP6AB116-qPCR-Reverse	GAAACGTGTATGGCCTTA
CYP314A1-qPCR-Forward	GCATGCAGGAGATCTTTC
CYP314A1-qPCR-Reverse	CGATCGCTTAGCAGGTAC
CYP9A209-qPCR-Forward	TGTCAAGCATCTACCTCC
CYP9A209-qPCR-Reverse	GTCTCGTACGAACACTATG
CYP6AB46-qPCR-Forward	TGTGGCGGTTGTTGCG
CYP6AB46-qPCR-Reverse	TGAAGTCGGTGGTGTAGCG
CYP324A1-qPCR-Forward	ATCCCGTTGGGGCTCTAA
CYP324A1-qPCR-Reverse	CAATTTTGCTGCCGTGAAG
CYP6AB3-qPCR-Forward	TTACCAGCCTTCGGGACG
CYP6AB3-qPCR-Reverse	CCCCGCAGCAAAGAACA
CYP4G8-qPCR-Forward	TGCCAGCCCTCATCCTCT
CYP4G8-qPCR-Reverse	GAACCTCCAGCGAAATCAAG
CYP6AB14-qPCR-Forward	AGTGCTCAGGGACCCAGATG
CYP6AB14-qPCR-Reverse	TCACCTTCAGCGACCAACAT
ACTIN-qPCR-Forward	CTTTCACCACCACCGCTG
ACTIN-qPCR-Reverse	CGCAAGATTCCATACCCA
GAPDH-qPCR-Forward	GGAAAGCTGACTGGTATGG
GAPDH-qPCR-Reverse	ACCTGGTCCTCGGTGTAG
CYP6AB196-RNAi-Forward	TAATACGACTCACTATAGGGAGAGACTTACTTGGAGTGGTG
CYP6AB196-RNAi-Reverse	TAATACGACTCACTATAGGGAGAGCACTATGGAAGACCTGG
CYP6AB116-RNAi-Forward	TAATACGACTCACTATAGGGAGAGAAATACCCAGATGAGAAGC
CYP6AB116-RNAi-Reverse	TAATACGACTCACTATAGGGAGAAGTGCTGAGATAACGGCT
CYP314A1-RNAi-Forward	TAATACGACTCACTATAGGGAGAGACAGATACGCTTCCACT
CYP314A1-RNAi-Reverse	TAATACGACTCACTATAGGGAGACTTCCATCAACTCAGAGACT
CYP9A209-RNAi-Forward	TAATACGACTCACTATAGGGAGACTACACTCAGTCCAGCTTTT
CYP9A209-RNAi-Reverse	TAATACGACTCACTATAGGGAGAGTTTCCTTTCTTAGCCTCCA
EGFP-RNAi-Forward	TAATACGACTCACTATAGGGAGACCTGAAGTTCATCTGCACCAC
EGFP-RNAi-Reverse	TAATACGACTCACTATAGGGAGACTCCAGCAGGACCATGTGATC

Sequence of T7 promoter was underlined.

Table S2. Number of sequences analyzed, estimated richness (ACE and Chao1), diversity index (Shannon and Simpson) and estimated sample Coverage for the different samples.

Table S2.

**Sample**	**Reads number**	**OUT**	**Ace**	**Chao**	**Shannon**	**Simpson**	**Coverage**

**AF3a**	**15963**	**131**	**350.98**	**233**	**0.64**	**0.80**	**0.9957**
**AF3b**	**15963**	**214**	**286.31**	**279.17**	**1.52**	**0.55**	**0.9957**
**AF3c**	**15963**	**341**	**388.44**	**399.16**	**2.57**	**0.26**	**0.9952**
**AF4a**	**15963**	**613**	**701.36**	**691.99**	**3.02**	**0.18**	**0.9909**
**AF4b**	**15963**	**297**	**321.41**	**319.5**	**2.73**	**0.17**	**0.9971**
**AF4c**	**15963**	**148**	**486.39**	**318**	**0.82**	**0.65**	**0.9947**
**AF5a**	**15963**	**286**	**327.16**	**339.05**	**1.61**	**0.56**	**0.9960**
**AF5b**	**15963**	**317**	**352.94**	**346.02**	**2.46**	**0.30**	**0.9962**
**AF5c**	**15963**	**93**	**441.93**	**264.11**	**0.35**	**0.90**	**0.9965**
**PS3a**	**15963**	**499**	**519.53**	**529.84**	**4.30**	**0.04**	**0.9967**
**PS3b**	**15963**	**319**	**402.73**	**385.23**	**2.50**	**0.24**	**0.9941**
**PS3c**	**15963**	**382**	**391.45**	**402.3**	**4.43**	**0.03**	**0.9982**
**PS4a**	**15963**	**230**	**293.26**	**279.91**	**2.00**	**0.32**	**0.9957**
**PS4b**	**15963**	**473**	**485.74**	**510**	**4.56**	**0.03**	**0.9976**
**PS4c**	**15963**	**326**	**359.46**	**367.59**	**3.35**	**0.10**	**0.9962**
**PS5a**	**15963**	**192**	**407.02**	**291.39**	**2.11**	**0.25**	**0.9951**
**PS5b**	**15963**	**407**	**447.12**	**448.90**	**3.81**	**0.05**	**0.9955**
**PS5c**	**15963**	**54**	**118.84**	**116**	**0.51**	**0.81**	**0.9980**
**AF_PS4a**	**15963**	**241**	**420.14**	**348.13**	**3.08**	**0.12**	**0.9949**
**AF_PS4b**	**15963**	**487**	**506.46**	**517.95**	**4.32**	**0.04**	**0.9969**
**AF_PS4c**	**15963**	**370**	**382.60**	**392.04**	**2.57**	**0.34**	**0.9978**
**AF_PS5a**	**15963**	**398**	**408.46**	**417.89**	**4.31**	**0.05**	**0.9982**
**AF_PS5b**	**15963**	**939**	**1114.76**	**1110.01**	**5.04**	**0.02**	**0.9856**
**AF_PS5c**	**15963**	**136**	**256.08**	**228.81**	**2.25**	**0.15**	**0.9966**
**PS_AF4a**	**15963**	**410**	**449.94**	**444.63**	**2.65**	**0.23**	**0.9954**
**PS_AF4b**	**15963**	**325**	**413.67**	**393.32**	**2.48**	**0.21**	**0.9938**
**PS_AF4c**	**15963**	**320**	**401.27**	**394.27**	**2.49**	**0.17**	**0.9939**
**PS_AF5a**	**15963**	**178**	**246.14**	**220.89**	**2.04**	**0.19**	**0.9960**
**PS_AF5b**	**15963**	**409**	**510.94**	**496.07**	**2.76**	**0.18**	**0.9925**
**PS_AF5c**	**15963**	**793**	**876.12**	**874.18**	**3.96**	**0.13**	**0.9907**
